# A coarse-to-fine approach to prostate boundary segmentation in ultrasound images

**DOI:** 10.1186/1475-925X-4-58

**Published:** 2005-10-11

**Authors:** Farhang Sahba, Hamid R Tizhoosh, Magdy M Salama

**Affiliations:** 1 Medical Instrument Analysis and Machine Intelligence Group, University of Waterloo, Waterloo, Canada; 2 Department of Systems Design Engineering, 200 University Avenue West, University of Waterloo, Waterloo, Ontario, N2L 3G1, Canada; 3 Department of Electrical and Computer Engineering, 200 University Avenue West, University of Waterloo, Waterloo, Ontario, N2L 3G1, Canada

## Abstract

**Background:**

In this paper a novel method for prostate segmentation in transrectal ultrasound images is presented.

**Methods:**

A segmentation procedure consisting of four main stages is proposed. In the first stage, a locally adaptive contrast enhancement method is used to generate a well-contrasted image. In the second stage, this enhanced image is thresholded to extract an area containing the prostate (or large portions of it). Morphological operators are then applied to obtain a point inside of this area. Afterwards, a Kalman estimator is employed to distinguish the boundary from irrelevant parts (usually caused by shadow) and generate a coarsely segmented version of the prostate. In the third stage, dilation and erosion operators are applied to extract outer and inner boundaries from the coarsely estimated version. Consequently, fuzzy membership functions describing regional and gray-level information are employed to selectively enhance the contrast within the prostate region. In the last stage, the prostate boundary is extracted using strong edges obtained from selectively enhanced image and information from the vicinity of the coarse estimation.

**Results:**

A total average similarity of 98.76%(± 0.68) with gold standards was achieved.

**Conclusion:**

The proposed approach represents a robust and accurate approach to prostate segmentation.

## 1 Introduction

Prostate cancer is one of the most frequently diagnosed forms of cancer in the male population and the second cancer-related cause of death for this group [1,2]. Ultrasound imaging is a widely used technology for prostate biopsy. The accurate detection of the prostate boundary in ultrasound images is crucial for some clinical applications, such as the accurate placement of the needles during the biopsy, accurate prostate volume measurement from multiple frames, constructing anatomical models used in treatment planning and estimation of tumor border. These images are the result of reflection, refraction and deflection of ultrasound beams from different types of tissues with different acoustic impedance [3]. However, in ultrasound images the contrast is usually low and the boundaries between the prostate and background are fuzzy. Also speckle and weak edges make the ultrasound images inherently difficult to segment. Furthermore, the quality of the image depends on the type and particular settings of the machine. All these factors make the analysis of ultrasound images challenging. But it still remains an important image modality for clinical applications and an automatic segmentation of these images is highly desirable. This work is organized as follows: In section II existing literature on prostate segmentation is briefly reviewed. The motivation for this work is summarized at the end of section II. Section III introduces the proposed approach and describes its main components in detail. Section IV validates the performance of the method via visual inspection and some quantitative measures. Section V concludes the work.

## 2 Related work

Currently, the prostate boundaries are generally extracted from TRUS images [3]. As previously mentioned, in TRUS images of the prostate, the signal-to-noise ratio is very low. Therefore, traditional edge detectors fail to extract the correct boundaries. Consequently, many methods have been introduced to facilitate more accurate and automatic or semi-automatic segmentation of the prostate boundaries from the ultrasound images.

Knoll *et al*. [4] considered deformable contours for prostate segmentation in medical images for both initialization and modeling. They have proposed a method based on a one-dimensional dyadic wavelet transform as a multiscale contour parameterization technique to constrain the shape of the prostate model.

Richard *et al*. [5] presented an algorithm which segments a set of parallel 2D images of the prostate into prostate and non-prostate regions to form a 3D image of the prostate. This texture-based algorithm is a pixel classifier based on four texture energy measures associated with each pixel in the image. Clustering techniques are then used to label each pixel in the image with the label of the most probable class.

Arnink *et al*. [6] described a method for determination of the contour of the prostate in ultrasound images. They have used an edge detection technique based on nonlinear Laplace filtering. The method then combines the information about edge location and strength to construct an edge intensity image. Finally, edges representing a boundary are selected and linked to build the final outline.

Ladak *et al*. [7,19] proposed an algorithm for semi-automatic segmentation of the prostate from 2D ultrasound images. The algorithm uses model-based initialization and a discrete dynamic contour. First, the user must select four points around the prostate. Then the outline of the prostate is estimated using cubic interpolation functions and shape information. Finally, the estimated contour is deformed automatically to better fit the image. This semi-automatic algorithm can segment a wide range of prostate images, but at least four initial points must be selected manually by the user (radiologist).

Prater *et al*. [8] presented a method for segmentation of the prostate in transrectal ultrasound images based on feed-forward neural networks. This method segments images to prostate and non-prostate regions. Three neural network architectures have been proposed. These networks are trained using a small portion of a training image segmented by an expert and then applied to the entire training image.

Wang *et al*. [30] presented two methods for semiautomatic three-dimensional (3-D) prostate boundary segmentation using 2-D ultrasound images. The segmentation process is initiated by manually placing four points on the boundary of a selected slice. Then an initial prostate boundary is determined. It is refined using the Discrete Dynamic Contour until it fit the actual prostate boundary. The remaining slices are then segmented by iteratively propagating the result to another slices and implementing the refinement.

Hu *et al*. [31] proposed an algorithm for semiautomatic segmentation of the prostate from 3D ultrasound images. In this method the authors use model-based initialization and mesh refinement using deformable models. Six points are required to initialize the outline of the prostate using shape information. The initial outline is then automatically deformed to better fit the prostate boundary.

Chiu emphet al. [32] introduced a semi-automatic segmentation algorithm based on the dyadic wavelet transform and the discrete dynamic contours. In this method first a spline interpolation is used to determine the initial contour based on four user-defined initial points. Then the discrete dynamic contour refines the initial contour based on the approximate coefficients and the wavelet coefficients generated using the dyadic wavelet transform. A selection rule is used as well to choose the best contour based.

Abolmaesumi *et al*. [33] used a segmentation technique to extract prostate contours from Transrectal Ultrasound images. In this method an Sticks filter is used to reduce the speckle. The problem is then discretized by projecting equispaced radii from an arbitrary seed point inside the prostate cavity towards its boundary. Candidate edge points obtained along each radius include the measurement points and some false returns. This modelling approach is used for prostate contour extraction.

Ghanei *et al*. [9] proposed a three-dimensional deformable surface model for prostate segmentation based on a discrete structure which is made from a set of vertices in the 3D space as triangle facets. The model converges from using a weighted sum of the internal and external forces. The model is initialized manually from a few human-drawn polygons drawn on different slices.

Pathak *et al*. [10] proposed an algorithm for guided edge delineation which provides automatic prostate edge detection as a visual guide to the observer. It is followed by manual editing. The edge detection algorithm contains three stages. First, the sticks algorithm is used to enhance contrast and reduce speckle in the image. Second, the resulting image is smoothed using an anisotropic diffusion filter. Finally, some basic prior knowledge of the prostate, such as shape and echo pattern, is used to detect the most probable edges which indicate the prostate shape. In the last stage, the information is integrated by using a manual linking procedure on the detected edges.

Shen *et al*. [11] introduced a statistical shape model to segment the prostate in transrectal ultrasound images. First, a Gabor filter bank is used in both multiple scales and multiple orientations to characterize the prostate boundaries. The Gabor features are reconstructed to be invariant to the rotation of the ultrasound probe. Then, a hierarchical deformation strategy is used. The model focuses on the similarity of different Gabor features at different deformation stages using a multiresolution technique. The authors have also introduced an adaptive focus deformable model, which uses the concept of an attribute vector [12].

In another work Gong *et al*. [28] presented an approach based on deformable models. In this technique, model initialization and constraining model evolution are based on prior knowledge about the prostate shape. The prostate shape has been modeled using deformable superellipses.

Betrouni *et al*. [29] discussed a method for the automatic segmentation of trans-abdominal ultrasound images of the prostate. In this method a filter is used to enhance the contours without changing the information in the image. Adaptive morphological and median filtering are employed to detect the noise-containing regions and smooth these areas. Then a heuristic optimization algorithm begins to search for the contour initialized from a prostate model.

Generally, prostate segmentation methods have limitations when the image contains shadows with similar gray level and texture attached to the prostate, and/or missing boundary segments. In these cases the segmentation error may increase considerably. Another obstacle may be the lack of a sufficient number of training (gold) samples if a learning technique is employed. Algorithms based on active contours have been quite successfully implemented with the major drawback that they depend on user interaction to determine the seed points (initial snake).

Based on an analysis of the existing literature a new approach should ideally possess certain properties:

• User interaction (e.g. defining seed points) may not be always desirable due to its drawbacks such as time consumption, human error etc. The new technique, therefore, should require a minimum level of user interaction.

• Providing a large number of training samples in medical environments is generally difficult, specially if the samples are being prepared by an expert. Hence, sample-based learning should be avoided.

• The approach must be robust with respect to the presence of noise and shadow.

In this paper, by introducing a multi-stage, coarse-to-fine approach, we establish a straightforward algorithm which attempts to satisfy the above conditions as much as possible. In this method some input parameters must be adjusted for series of images (i.e. images captured with a certain equipment setting).

## 3 Proposed approach

**Figure 1:**
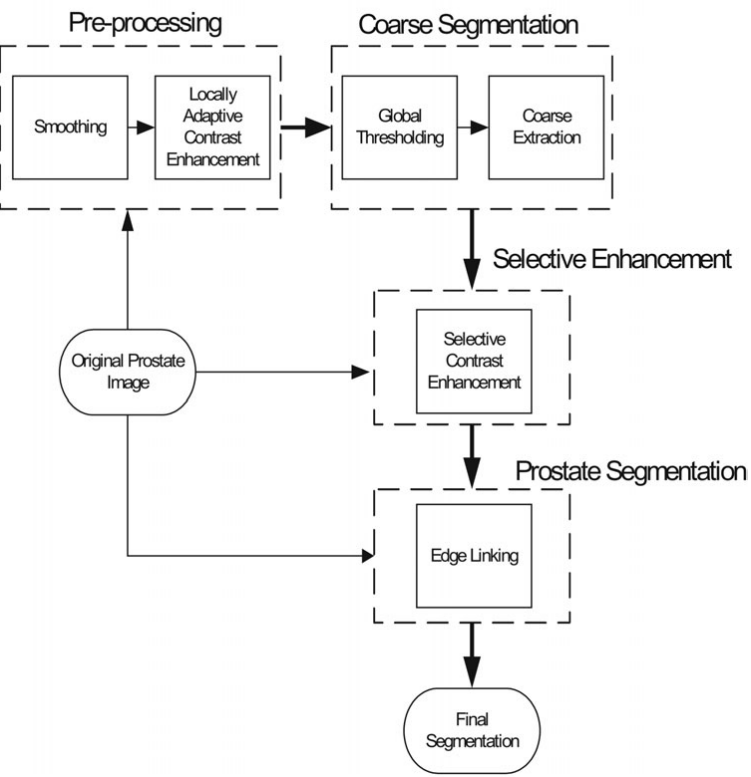
Schematic diagram of the proposed approach.

In this section, we will present a new region- and grayscale-based approach to prostate segmentation in ultrasound images. The proposed approach contains four main stages (see Fig. 1):

• **Pre-Processing: **After smoothing, using a locally-adaptive contrast enhancement technique, a primary version of the image which has sufficient contrast across the image is produced (see section 3.1).

• **Coarse Segmentation: **The obtained image is thresholded and some morphological operators are applied until an isolated object related to the prostate is produced. The central point of this object can be considered as the central point of the prostate. Subsequently, using a Kalman estimator, a coarse estimation for the prostate boundary is obtained. This coarse estimation is not accurate, but we just use it as input for the next stage (see section 3.2).

• **Selective Enhancement: **Using coarse estimation, one eroded and one dilated version can be produced. These are used to establish regional fuzzy membership functions. By defining a fuzzy inference system based on the produced membership functions, a selective contrast enhancement can be obtained in the area of the prostate (see section 3.3).

• **Prostate Segmentation: **Finally, the algorithm finds potential boundary pieces and extracts the prostate boundary (see section 3.4).

In the following sections, each stage of the proposed approach will be described in detail.

### 3.1 Pre-processing

**Figure 2:**
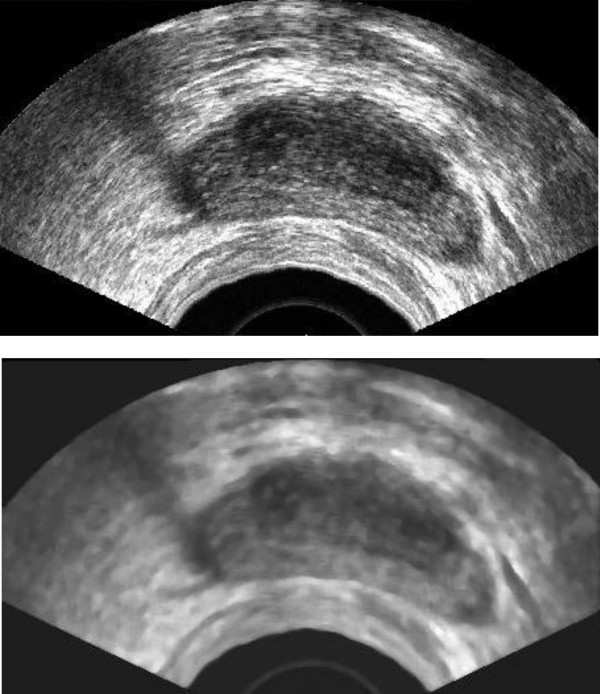
Top: Original image, Bottom: Smoothed image using a median filter 9 × 9.

Transrectal ultrasound images are heavily corrupted with noise. Since we just need a rough estimation for the next stage, we can remove the noise using a median filter (7 × 7 or 9 × 9). It should be mentioned that this smoothed version is only used for the next step. For the final segmentation we use the "original" ultrasound image. Therefore, the median filter does not manipulate the edge information needed for the final segmentation. Fig. 2 shows the original TRUS and its smoothed version.

In order to enhance the contrast of the image, a locally-adaptive method based on fuzzy sets has been used to create a primary version which has enough contrast [13,14,25].

In a fuzzy approach, an image can be presented as an array of fuzzy singletons (a membership function with only one supporting point). Each of these singletons has a value that denotes the membership of each pixel to a predefined image property. The properties can be defined globally for the whole image or locally for sub-images [13]. In the case of contrast enhancement using fuzzy techniques, some parameters of spatial domain, such as minimum gray-level *g*_*min *_or maximum gray-level *g*_*max*_, are needed. In a global method, finding those parameters is simple, but if we want to enhance the image in a locally approach, then we must calculate these parameters for each local neighborhood to adjust the membership function. Tizhoosh *et al*. [13] have presented a locally adaptive approach to find the parameters for some support points for an *M *× *N*-image and interpolate these values to obtain corresponding values for each pixel. It is clear that these interpolated parameters are not precise, but because the concept of fuzziness is incorporated, the input data have not to be exact [13,14]. In the method proposed in [13], first the image is divided into *M*_*S *_rows and *N*_*S*_. This leads to a matrix with the size of *M*_*S *_× *N*_*S*_. Each element in this matrix corresponds to a supporting point for the image. A window around each supporting point is considered to find the local information for that point. In a general case the size of window around each supporting point depends on the degree of homogeneity or edginess of local pixels in the corresponding sub-image. Small window sizes do not only increase the computational cost but also sometimes fail to capture correct information due to noise sensitivity. On the other hand, with large window sizes, values obtained by interpolated parameters lose their accuracy. In our application we use constant window sizes around each supporting point to find the local information. Just to avoid loss of information during the interpolation, the size of windows is chosen in a way that they have enough overlap with each other. Using the local windows we can extract local parameters, like *g*_*min*,*local *_and *g*_*max*,*local*_. Applying a 2-D interpolation function (e.g. linear or cubic), the membership function parameters can be calculated for the whole image [13].

**Figure 3:**
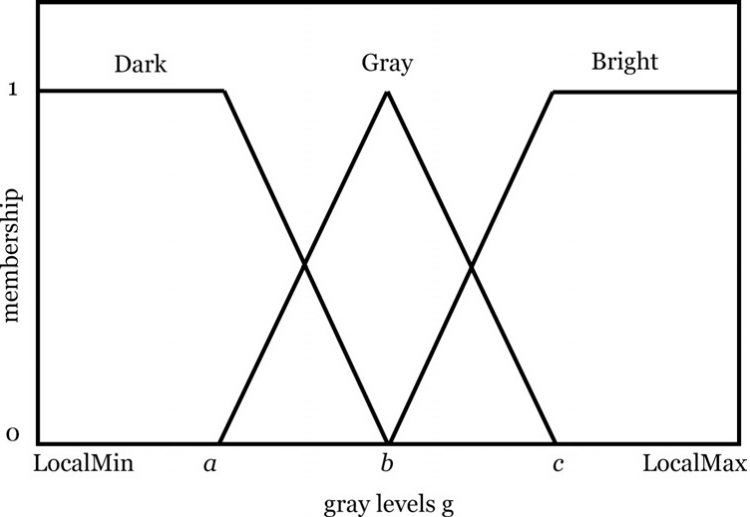
Input membership function for locally adaptive contrast enhancement. It represents three terms for the fuzzy set "pixel intensity". Generally *b *is in the middle value of LocalMin and LocalMax, *a *is the middle values of LocalMin and *b*, and *c *is the middle value of LocalMax and *b*.

To apply this method in our case we have used a maximum window size of 40 × 40 and a minimum window size of 20 × 20, both determined heuristically. The algorithm calculates a proper window size between these two values using fuzzy rules. Among methods introduced in [13] we have employed a fuzzy rule-based approach to enhance the contrast for the entire image [13-16]. The input membership functions used in this fuzzy rule-based approach are shown in Fig. 3.

For the output membership function we have used three singleton values *g_S1 _*= 10, *g_S2 _*= 100 and *g_S3 _*= 255.

**Figure 4:**
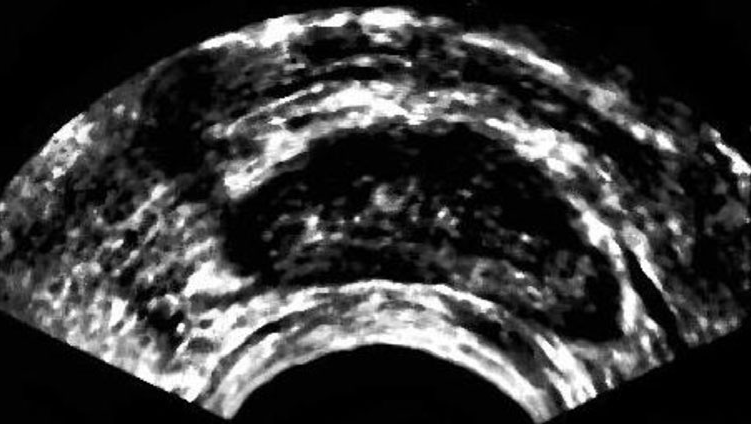
Primary high contrast version of the smoothed image from Fig. 2.

**Figure 5:**
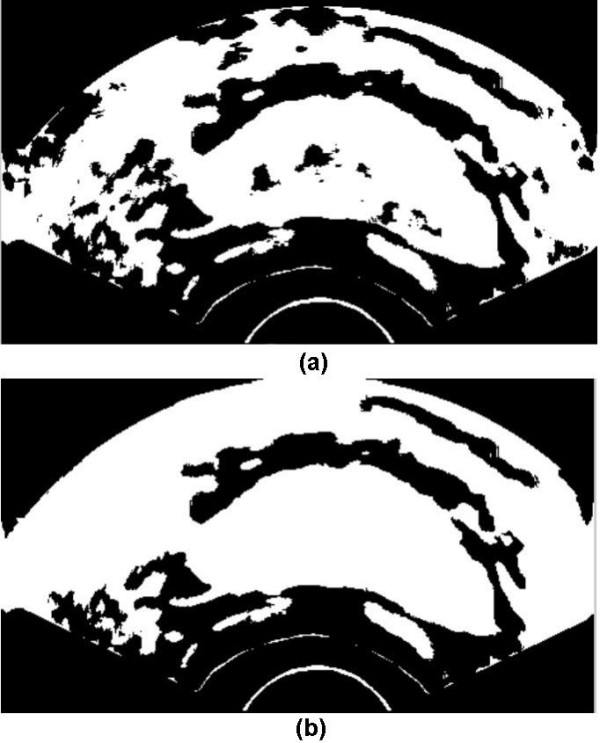
(a): The result of global thresholding on primary high contrast version in Fig. 4, (b): Filled version of the global thresholded image.

The result of applying the above method on the smoothed image followed by global thresholding on the enhanced image are shown in Fig. 4 and 5, respectively. As we can see in Fig. 4, applying the primary contrast enhancement makes a significant gray level difference between the dark areas, including prostate, and bright gaps around the prostate.

For global thresholding, all gray levels *g *less than a threshold value *T *are set to zero and those greater than this threshold are set to 255 such that a binary image with black and white values *g' *is obtained. Fig. 5(a) shows the result of applying global thresholding to the image in Fig. 4. The main advantage of applying the primary contrast enhancement is that the bright gaps produced around the prostate have distinctly large gray level intensities. Consequently, an image with a bimodal histogram is produced. *T *is chosen as a middle value between two peaks in the histogram and for a large category of ultrasound images, good performance of global thresholding can be expected.

### 3.2 Coarse estimation

This section demonstrates how a central point inside the prostate can be found. We will see that using this point and some other information how a Kalman estimator achieves a coarse estimation of the prostate boundary.

The binary image obtained after thresholding contains several black holes (Fig 5(a)). These holes can be filled such that a completely white region is archived (Fig 5(b)) [17]. Applying the primary contrast enhancement stage ensures the extraction of a white area corresponding to the prostate in the thresholded image. This area must be isolated from other white regions. One straightforward method uses the morphological opening [17]. This operator must be applied with a large structuring element to isolate the object corresponding to the prostate. Considering the geometrical location and the measurement of area, the isolated object with high likelihood to be part of the prostate can be distinguished. A point *O *inside the prostate is required to perform the next stage. This point can be simply obtained by finding the centroid point of this isolated object.

Now we want to find a coarse estimation of the prostate boundary. This can be solved using a Kalman filter [18] for tracing the edges. We can use some properties of this filter to extract the valid data from the thresholded image and remove the irrelevant parts. To implement such a system we interpret the problem of edge tracing as a dynamic tracking system. In this system the pixels located on the edge of an object of interest are used as the input (measurement data) for the tracking filter. Using such a system the Kalman filter can track a trajectory along the border of the object. Each new pixel along the border brings updated information for the current and future position. We assume each pixel along the border to be in a dynamic movement. For this movement we can consider the position and velocity as the variables which describe the state of the system. Using a Kalman filter we can estimate the position which is actually the border of the object of interest. In fact we use a tracking system to follow the border. In a 2D image we have two variables for position and two variables for velocity. Because of the shape of the prostate it is more efficient to represent the state variables in the form of relative polar coordinates. We can use the internal point *O *to define such state variables and consider the following state vector:



where *r *is the distance between the internal point *O*(*x*_*c*_, *y*_*c*_) and pixels (*x*_*p*_, *y*_*p*_) located on the border of the prostate and *θ *is the angle between the vertical axis and . Hence, the following equations can be considered for r, *θ*,  and :









where ,  are the radial and angular velocity, respectively. Using the above state vector we represent the sequential pixels on the border of the object of interest (corresponding to the movement of vehicle on a path) by radial and angular position and velocity. Kalman filter considers a discrete dynamic model contains state and measurement equations:

**x**_*k *_= **A**_*k*-1_**x**_*k*-1 _+ **B**_*k*-1_**W**_*k*-1_,     (6)

**Z**_*k *_= **H**_*k*_**x**_*k *_+ **V**_*k*_,     (7)

where **x **is the state vector in equation 1. Other components are defined as follows:







where **W**_*k *_~ *N*(0,*Q*_*k*_) and **V**_*k *_~ *N*(0, *R*_*k*_) are the process and measurement noise, respectively. *T *is the interval which represents the changes in the state and measurement equations. The value of *T *does not affect the final result and therefore we can choose it as *T *= l for simplicity. *R *and *Q *are diagonal matrices. The values in these matrixes are the measurement and process noise covariance, respectively. In the area that border is changing smoothly the measurement data is placed inside the association gate and the value of measurement noise *R *should be small. In the case when there is no data inside the association gate we cannot be ensure about the validity of measurement data. Therefore the value of measurement noise *R *should be large. The large and small values for the measurement noise depend on the quality of image and can be adjusted between 5 to 100. The value of process noise in matrix *Q *simulates the small variation around the estimated point. For process noise we can consider a value between 4 to 8. In the discrete dynamic equations the accelerations in the radius and angle (the changes of radial and angular velocities) is modeled as a zero-mean, white, Gaussian noise **W**. Also the measurement data **Z**_*k *_which is the location of boundary pixels in the form of *r *and *θ *is assumed to be a noisy version of the actual position of the boundary. Kalman filtering is done using prediction and update steps. The prediction equations include

**x**_*k*|*k*-1 _= **A**_*k*-1_**x**_*k*-1|*k*-1_,     (11)



whereas update equations can be given as follows



**K**_*k*-1 _= **P**_*k*|*k*-1_**H**_*k*-1_**S**^-1^,     (14)

**x**_*k*-1|*k*-1 _= **x**_*k*-1|*k*-2 _+ **K**_*k*-1_[**Z**_*k*-1 _- **H**_*k*-1_**x**_*k*-1|*k*-2_],     (15)

where **P **is error covariance matrix, **K **is kalman gain and **S **is innovation covariance matrix. For calculation of the coarse version, the Kalman filter receives its initial data from the nearest point placed on the vertical axis and on the top of the internal point *O*. Then it starts the estimation using the data located on the border of the object in the thresholded image. In each sequential iteration, the points along the prostate border can be used as measured data and the Kalman filter estimates the next *r *and *θ*. These predicted values determine a pixel as the next pixel on the border. Also it predicts  and  for the next iteration. When we go to the next iteration the new pixel on the border is the new measured data for the filter. This data is compared to the predicted position from the previous iteration. If there is sufficient correlation between them the measured data is incorporated to update the filter state, otherwise the prediction point is considered as measured point and after updating the filter starts the next iteration. To measure the correlation we implement an association process between the predicted and measured data. For this association process we use a gate, the so-called "association gate" around the predicted pixel. Only the pixels located on the border and inside of this gate are considered as valid measured data for updating the filter. For good performance, the association gate must be adaptive. This means that the size of this gate must be varied based on the covariance of the Kalman filter. The gate must maximize the presence of valid data and minimize invalid data. To find such a gate, the probability ratio function, *L*, at the *k*^*th *^iteration must be maximized [18]:



where *p*_1_(*k*) and *p*_0_(*k*) are the probabilities of the presence of valid data and invalid data in the gate, respectively. This prompts us to check the value of a statistical distance *d *with respect to a threshold *D *as follows [18]:

*d*^2 ^≤ *D*,     (17)

with

**Figure 6:**
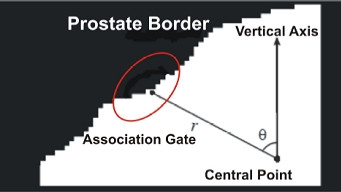
local polar coordinate schema and elliptical gating for extracting the coarse estimation.

*d*^2 ^= **l**^T^**P**^-1^**l**,     (18)

where **l **is the vector of geometrical distance between the predicted point and an arbitrary point *l *= (*l*1, *l*2), and **P **is the covariance matrix of the Kalman filter. In two dimensional problems, equation 18 is reduced to the following form:



where *a, b, c *and *e *are the values of covariance matrix. This is an equation for the points inside of an ellipse. The value of *D *is generally a constant between 1 to 10. In the case when there is no data inside the association gate we need to capture the data. Therefore, the value of *D *should gradually increase. Fig. 6 illustrates this local polar coordinate schema and elliptical gating.

**Figure 7:**
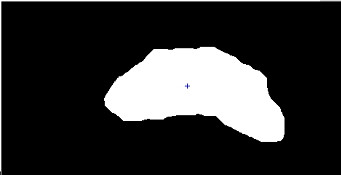
Isolated object corresponding to the prostate and its center.

When the border reaches a shadow, or a missing boundary segment is encountered, then there is an abrupt change in the pixel path. These sharp changes are considered as new paths for tracking process. After a few iterations Kalman filter detects that such cases do not belong to the true path because the data do not correlate with the followed path. The filter adaptively enlarges the association gate until it again captures the true data on the border which have enough correlation with the prediction. Using the association technique the data belonging to shadows and missing segments on the prostate border are detected and eliminated. During the process, Kalman estimator follows the prostate border variations in a coarse manner and irrelevant parts are isolated. Fig. 7 shows this coarse estimation for the prostate.

In the most parts, the gray level difference produced by primary contrast enhancement makes the prostate border distinguishable after thresholding. Applying the Kalman estimator ensures that the prostate is isolated from any irrelevant parts and a coarse estimation is achieved. An interesting result is that if the quality of ultrasound images are good enough there is no irrelevant parts attached to the prostate and the boundary can be visible after primary contrast enhancement and thresholding. In these cases we do not need to employ the Kalman filter and coarse estimation may even be used as the final segmented image.

The quality of this coarse estimation directly depends on the quality of the ultrasound image in terms of original contrast, the presence and intensity of shadow, the noise and so on. For all tested images the extracted coarse estimation had sufficient accuracy to be passed to subsequent stages.

### 3.3 Selective enhancement

In this section a new regional approach is introduced to increase the prostate contrast. This will amplify the strength of the outer prostate edges.

The previous section contained some techniques to find a coarse estimation. Using the boundary generated by the coarse estimation, two contours can be obtained such that the true boundary is ideally located between them. In our approach, these are called inner and outer contours. For extracting the inner contour, an erosion operator with a disk-shaped structuring element can be employed on the coarse estimation. With the same structuring element and using a dilation operator, the outer contour can be obtained as well. These two contours are employed as points of departure to define a membership function.

**Figure 8:**
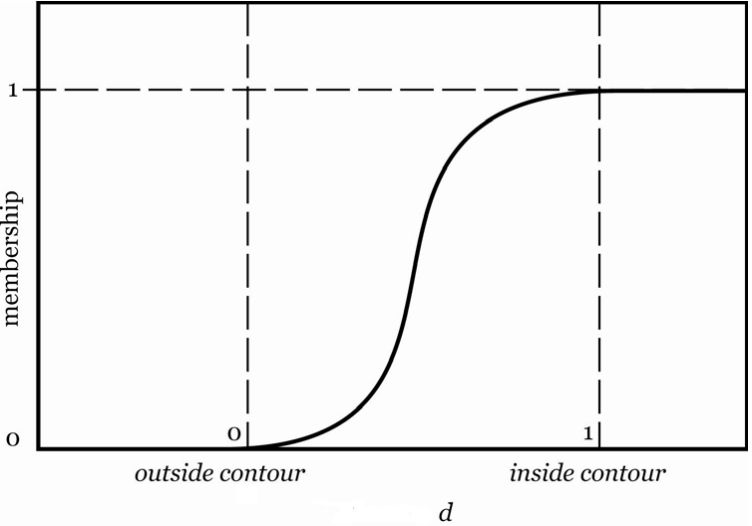
the membership function *μ*_*location *_based on pixel position. In this Figure  and *d*_*inside*_and *d*_*outside *_are the shortest euclidian distance to the outside and inside contours respectively.

This membership function determines to what degree does a pixel belong to the prostate. Fig. 8 illustrates the membership function *μ*_*location *_based on pixel position. Because the boundary of the coarse estimation is extracted from the object corresponding to the prostate, it can be assumed that the true edges of the prostate are located around this boundary. If the pixel is located outside of the outer contour, the membership value is 0, if it is inside of the inner contour, the membership value is 1. For the pixels in between, the nearest distance from the inner and outer contours is determined and the value of membership is calculated. Because the pixels located between the inside contour and the coarse estimation boundary most likely belong to the prostate, the membership function has more emphasis on this interval.

Similarly, this function has less emphasis for the pixels located between the coarse estimation boundary and the outer contour because the pixels located around the outer contour most likely do not belong to the prostate. In fact, the membership value *μ *depends on the following equation:



where *α *= 6, *β *= 0.5 (heuristically selected parameters for the sigmoidal function) and the value of *d *is calculated as follows



where *d*_*inside *_and *d*_*outside *_are the shortest euclidian distance of arbitrary points to the outer and inner contours, respectively. For the establishment of a fuzzy inference system, we need to define appropriate fuzzy rules [13,14]. The area between the two contours is important for the extraction of the prostate boundary. Since we assume that there is generally a dark to light transition from the inside to outside of the prostate, any algorithm improving the strength of edges in this area is useful. A straightforward method is to make the dark and gray pixels darker and bright pixels brighter. This can increase the strength of the edges around the prostate boundary. In addition, as previously discussed, we want to enhance the contrast just for the area within the prostate. Therefore, simple rules can be defined as follows:

• **IF **the pixel *does not belong *to the prostate, **THEN **leave it *unchanged*

• **IF **the pixel *belongs *to the prostate **AND **is *dark*, **THEN **make it *darker*

• **IF **the pixel *belongs *to the prostate **AND **is *gray*, **THEN **make it *dark*

• **IF **the pixel *belongs *to the prostate **AND **is *bright*, **THEN **make it *brighter*

**Figure 9:**
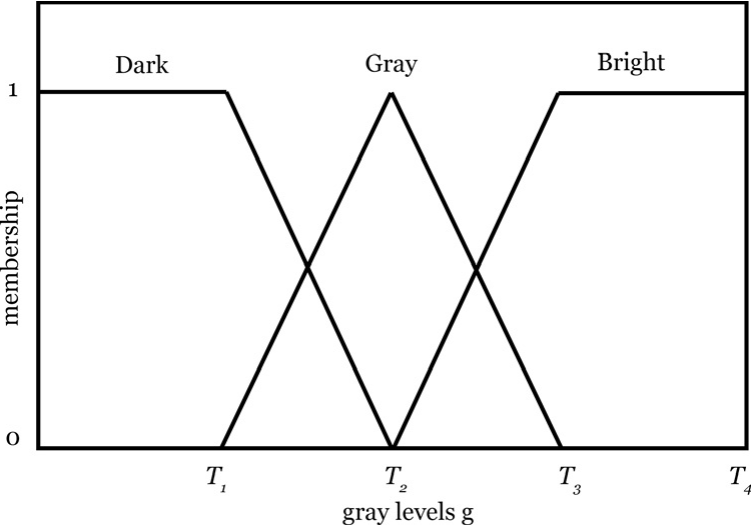
Membership functions for input gray level values.

The last rule is mainly designed to enhance the brighter boundary pixels (bright pixels within the prostate are not relevant at this stage). The membership function for input gray levels is shown in Fig. 9. In this figure *T*_4 _is the brightest gray level in the image. *T*_1_, *T*_2 _and *T*_3 _can be calculated based on local information, but for simplicity we have used the values 25, 50 and 80, respectively. For the output membership function, fuzzy singletons with the values *G*_*S*1_*= *1, *G*_*S*2 _= 64 and *G*_*S*3_*= *255 are defined empirically.

**Figure 10:**
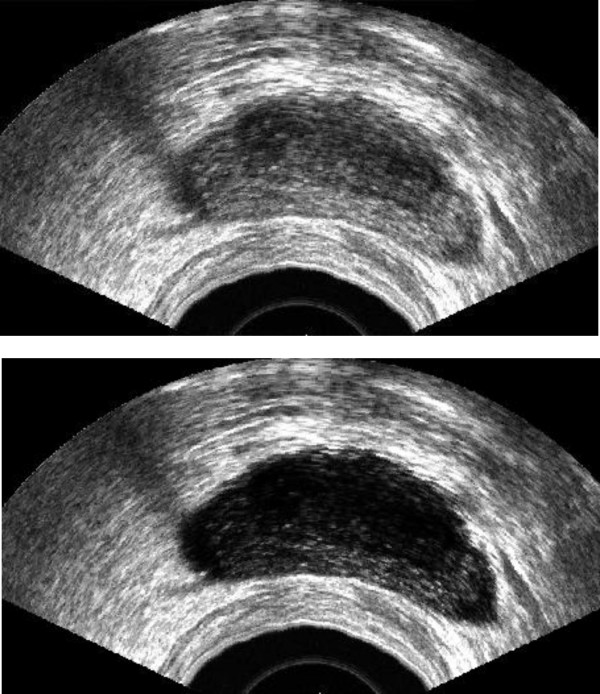
Top: Original image. Bottom: Enhanced Image using proposed fuzzy inference.

Using these rules we enhance the contrast of the image not only based on gray level values but also based on the location of each pixel. The result is shown in Fig. 10. It clearly demonstrates that the result is an image with higher contrast in the prostate area.

## 3.4 Final segmentation

**Figure 11:**
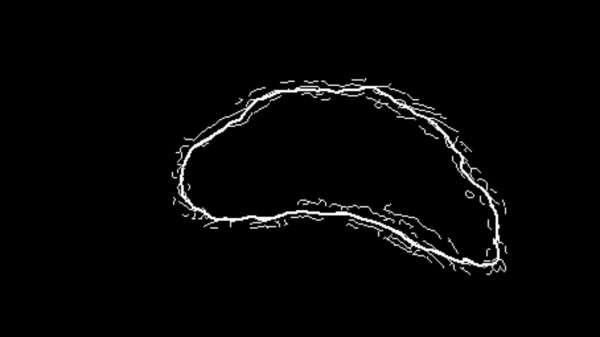
The result of applying Canny edge detection on the enhanced image (Fig. 10). Solid line is the coarse estimation outline.

In the previous section we achieved an image with a highly contrasted prostate area and almost no changes in other regions such that strong edges were created in the boundaries of the prostate. Also we know that the true boundary is located between the inner and outer contours and somewhere most likely close to the coarse estimation. Therefore, we must first detect the edges located between these two contours. For this purpose, a Canny edge detector is used [21]. Fig. 11 shows the result for the area between two contours. This image contains many potential boundary pieces. Among these pieces, those with higher likelihood of being a true edge should be considered. We start from a point above the internal point *O*, traverse along the coarse boundary and consider small pieces (3 to 5 pixels) on this boundary, sequentially. For each edge segment on the coarse boundary there are several edge pieces. We use three criteria to extract the final edges:

1. The average distance  of *N *candidate pixels in the vicinity of the coarse boundary – we can calculate the vicinity distance by the following equation:



where *d*_*i *_is the distance of *i*^*th *^pixel with respect to the boundary of the coarse estimation.

2. The value of the gradient – In the preceding section, we enhanced the contrast of the prostate area. This increases the local gradient values of the pixels located on the true boundary pieces. The local gradient value in 3 × 3 neighborhoods is determined as:



where *G*_*x *_and *G*_*y *_are the gradient values in *x *and *y *directions, respectively.

3. The angle of edge pieces with respect to the coarse boundary – we can calculate the absolute value of angle between the coarse boundary piece and edge piece. This value can vary from 0 to *π*/2. Zero radians reflects the most and *π*/2 the least compatibility.

After these criteria are considered in the presented priority order, the final boundaries must be extracted from potential pieces. Using the above criteria we must extract the edge pieces which have the greatest likelihood of being a true edge. Among all pieces, a piece with minimum distance, maximum gradient, or minimum angle with respect to the boundary of the coarse estimation should be chosen. If we consider the border of the coarse version where there was no shadow and missing boundary segment, the information of the coarse version is more reliable. Therefore, in these parts our criteria for choosing an edge piece is the distance of that piece with respect to the coarse version. If the distances are equal, then the angles of pieces are considered. If the angles are equal as well, then the gradient values are compared. But in the parts where the Kalman filter has estimated the border of the coarse estimation the strengths of the edge pieces (using gradient values) are the only criteria for the selection. Subsequently, the algorithm goes directly to the next piece and continues the procedure until the complete prostate outline is achieved. For the coarse boundary pieces, if there is no edge piece around them we can continue the edge extracted in the previous coarse boundary piece so that it has the minimum angle with respect to the coarse estimation. A straightforward way to fill the gaps between adjacent pieces is to fill them by straight lines. Finally, we can again apply a Kalman filter to smooth these edges and make a consistent outline for the prostate to achieve the final result. This smoothing filter is just like what we used to find the coarse estimation except that it uses all final data on the prostate border.

**Figure 12:**
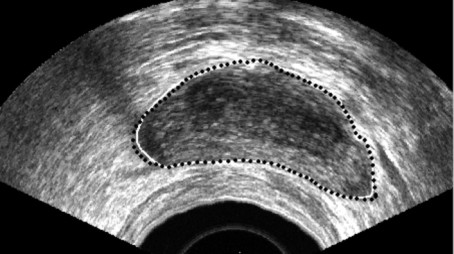
Automatic and manual boundaries are shown on the original images. Solid lines are manually segmented images and dash lines are the result of proposed algorithm.

**Figure 13:**
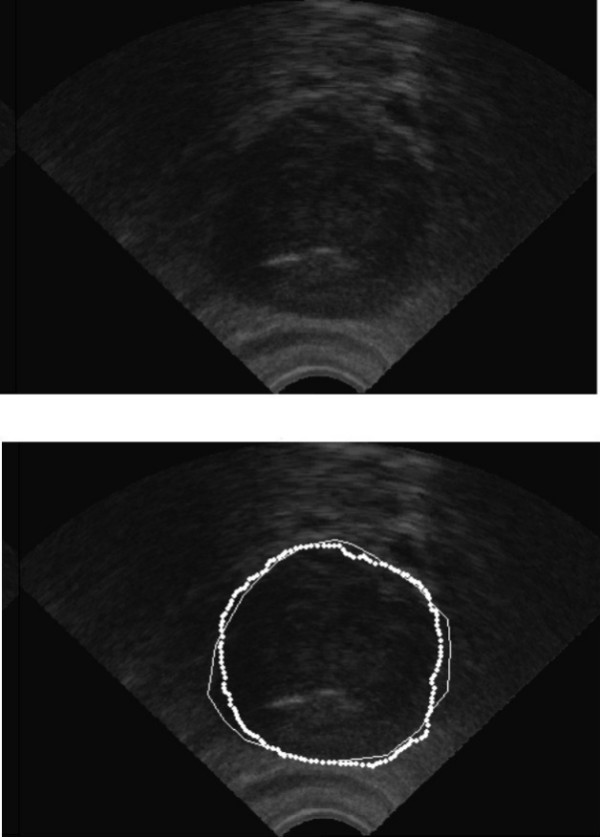
A low quality TRUS image and the result of automatic and manual boundaries shown on the original images. Solid lines are manually segmented images and dash lines are the result of proposed algorithm.

A sample result of employing the proposed approach on the extracted edge is shown in Fig. 12. This is the final result for the prostate segmentation. This figure shows the outline obtained manually by a radiologist (solid line). Visually, the difference between the two contours is negligible. To evaluate the algorithm for a low quality case, we have applied the proposed method in the image shown in Fig. 13. As we can see, the segmentation in the strongly shadowed areas have some error but in the other areas, the result is almost the same as manual segmentation.

## 4 Experiments and results

Other algorithms mentioned in section 2 have used different numbers of ultrasound images (from 8 [11] to 90 [7,19]) to validate their approaches. They have compared the algorithm-based segmentations with the manual segmentations (as a gold standard). We have selected the images to include regular and difficult cases and examined 42 different TRUS images. The images were noisy, with low contrast and shadow effects. The results of the proposed method have been evaluated by comparing the algorithm-based segmentation and the manual segmentation (gold standards).

As it was mentioned throughout the paper some parameters need to be adjusted in this algorithm. But for a set of similar TRUS images (i.e. images captured with a certain machine setting), most of them do not require any change. In the conducted experiments, the following parameter configurations have been used:

• The size of the median filter used for smoothing was 7 × 7.

• The structuring element used in the opening procedure was a disk with diameter 15.

For quantitative evaluation, we have used the following error measures to validate the performance of our segmentation compared to the manual segmentation by a radiologist for those images [22]:

**Distance ***δ *= Average Euclidean distance (in pixels) between the algorithm-based segmentation and the manual segmentation. For each pixel the distance is defined as the shortest Euclidean distance between that pixel and the pixels located on the other contour.

**Area ***E*_*A *_= Area error (%) = 100.,

where *S*_*Man *_is the area of the manual segmentation and *S*_*Alg *_is the area of the algorithm-based segmentation.

**Similarity **In addition, we have used a *similarity measure*, *η*, based on the misclassification rate as a more general criterion in image segmentation [23,24]:



where *B*_*0 *_and *F*_*0 *_denote the background and foreground of the original image (manually segmented), *B*_*T *_and *F*_*T *_denote the background and foreground area pixel in the result image, and |.| is the set cardinality.

Table I summarizes the results along with the average, *m*, and standard deviation, *σ*, for the three quantitative measures for all 42 test images.

**Table 1:** Quantitative evaluation of proposed approach in comparison with manual segmentation (gold standard). The average *m *and deviation *σ *for three performance measures are calculated for 42 test images and their corresponding gold standards.

Number	*δ*	*E*_*A*_	*η*
*m*	**3.67**	**5.62**	**98.76**
*σ*	**1.08**	**2.98**	**0.68**

Considering the quality of the images in terms of the shadow effect, the quantitative results are promising. From the results, it can be seen that in the areas that there is no shadow the proposed method is able to deliver very accurate results. In the areas, in which strong shadows cover the prostate the result of the algorithm is still acceptable. Of course, in these areas error is increased because the gray level and the texture of the shadow is very similar to those of the prostate. Qualitatively, the difference between the algorithm-based segmentations and the manual segmentations is not considerable. The proposed approach, implemented in MatLab, needs (in average) less than 10*s *to segment the prostate using a 2.8 *GHz *Pentium IV. However, this time measurement is based on experimental setup. It can be expected that a considerable speedup can still be achieved if the algorithm is implemented and optimized in realtime platforms such as C++.

## 5 Conclusion

A novel approach to prostate segmentation containing a coarse estimation and a new selective fuzzy contrast enhancement model has been presented in this paper. Because of the characteristics of the ultrasound images, we first smooth the original image using two filters. This smoothed image is enhanced using a locally-adaptive contrast technique. The output image has large gaps with high intensity around the prostate. Using global thresholding and morphological operators, an isolated object containing the prostate was obtained. The center of this object was considered as an internal point of the prostate. A Kalman estimator with the polar coordinates was implemented to find a coarse estimation of the prostate border. Using erosion and dilation of this estimation, inner and outer contours were obtained. A fuzzy inference system based on these regional contours and spatial gray level information was designed to selectively enhance the contrast of original image within the prostate region. The output of this fuzzy enhancement system provided an image with high contrast and strong edges on the prostate borders. Finally, the edges between inner and outer contours were extracted. In order to correctly recognize the prostate boundaries, potential boundary pieces were marked and based on pixels gradients, the vicinity and angle relative to the coarse estimation boundary, the final segmentation was achieved. The proposed approach has been examined for typical TRUS images. In comparison with manually segmented images, the experimental results show that our approach can segment the prostate boundary accurately. A total average similarity of 98.76%(± 0.68) with the gold standards was achieved. The test images contain not only regular but also noisy, low-contrasted and shadowy cases. In this approach we have designed a straightforward and fast algorithm with minimum level of user interaction. It also does not need training samples for implementation. More samples containing the manual prostate segmentation by radiologists are desired in order to verify the segmentation accuracy more reliably. In addition, by using an adaptive approach for noise reduction, we can improve the image quality, especially the prostate edges. This can give us better results in subsequent stages. In addition, developing of a similar technique for 3D prostate segmentation can be a subject for further work.

## 6 Authors' contributions

Farhang Sahba developed the main parts of the approach. Hamid R. Tizhoosh provided guidance and was involved in the theoretical aspects of algorithm development. Magdy M. Salama provided guidance. All authors read and approved the final manuscript.
